# Public acceptability of public health policy to improve population health: A population‐based survey

**DOI:** 10.1111/hex.13041

**Published:** 2020-04-24

**Authors:** Catherine A. Sharp, Mark A. Bellis, Karen Hughes, Kat Ford, Lisa C. G. Di Lemma

**Affiliations:** ^1^ Public Health Collaborating Unit School of Health Sciences Bangor University Wrexham UK; ^2^ Policy and International Health Directorate World Health Organization Collaborating Centre on Investment for Health and Well‐being Public Health Wales Wrexham UK; ^3^ Faculty of Health and Social Care University of Chester Chester UK

**Keywords:** alcohol, diet, general health, national survey, physical activity, policy, public acceptance, public health, smoking, well‐being

## Abstract

**Background:**

For public health policies to be effective, it is critical that they are acceptable to the public as acceptance levels impact success rate.

**Objective:**

To explore public acceptance of public health statements and examine differences in acceptability across socio‐demographics, health behaviours (physical activity, diet, binge drinking and smoking), health status and well‐being.

**Method:**

A cross‐sectional survey was conducted with a nationally representative sample (N = 1001) using a random stratified sampling method. Face‐to‐face interviews were conducted at homes of residents in Wales aged 16+ years. Individuals reported whether they agreed, had no opinion, or disagreed with 12 public health statements.

**Results:**

More than half of the sample were supportive of 10 out of 12 statements. The three statements with the greatest support (>80% agreement) reflected the importance of: a safe and loving childhood to becoming a healthy adult, schools teaching about health, and healthier foods costing less. Individuals who engaged in unhealthy behaviours were less likely to agree with some of the statements (eg 39.8% of binge drinkers agreed alcohol adverts should be banned compared to 57.6% of those who never binge drink; *P* < .001).

**Conclusions:**

Findings show an appetite for public health policies among the majority of the public. The relationship between supporting policies and engaging in healthy behaviours suggests a feedback loop that is potentially capable of shifting both public opinion and the opportunities for policy intervention. If a nation becomes healthier, this could illicit greater support for stronger policies which could encourage more people to move in a healthier direction.

## 1 INTRODUCTION

Public health literature has extensively examined the environmental and social determinants of health, and has identified the substantial contributions that inequality and lifestyle choices (eg diet, physical activity and smoking habits) make to population health and well‐being.^1^, ^2^, ^3^ Equally, an expanding evidence base shows that health inequalities and pressures on health services can be reduced through the implementation of effective public health policies, including supporting early childhood development, poverty alleviation, progressive taxation, restricted advertising, sustained public health messaging and improved access to health care.^4^ Investing in such solutions to improve health and well‐being is an increasing strategic priority globally^5^, ^6^ as countries strive to reach the 2030 Agenda for Sustainable Development and its Sustainable Development Goals (SDGs).^5^ Effective public health policies are required to help achieve these goals, and policies must invest across the life‐course and empower the public to take charge of their health.^4^

For public health policies to be effective and sustainable, it is critical that they are acceptable to the public. Despite this, health policy discussions can often occur in the absence of knowledge on public opinions on such policies or can be dominated by the views of patients rather than the public. The degree to which the public accepts a policy impacts its chances of success,^7^ and public involvement in the policy development process can increase such acceptance.^8^ Consequently, the importance of public involvement and an awareness of public opinion in policy making is increasingly recognized.^9^ A growing body of research linking public opinions with a range of governmental and policy agendas shows some congruence,^10^, ^11^, ^12^ and this can be stronger when public opinion is considered in the early stages of the decision‐making process.^13^ However, public opinions, expectations and demands are not always aligned with the priorities and resource allocation set by policy‐makers.^14^, ^15^ Further, views can differ across population groups based on their experiences.^16^ Thus, it is important to understand which public health policies are most likely to gain public support and which sections of the public may require more advocacy for public health policy and interventions.

Since the 1930s, politicians, researchers and the media have been using a variety of methods (eg face‐to‐face, telephone or online surveys; citizen juries or discussion groups)^17^ to measure public opinion across a wide range of topics.^7^, ^18^, ^19^, ^20^, ^21^ Internationally, however, while research has explored the public acceptability of different mechanisms of delivering policy messages,^22^, ^23^ relatively few nationally representative public opinion surveys have attempted to identify public acceptance of different health priorities and policies.^9^, ^14^, ^24^, ^26^ Furthermore, while some studies have examined differences in public acceptance of public health policies by socio‐demographic levels, less have explored differences according to current health status or health‐related behaviours. In 2017, a Canadian survey which focused on health inequalities^21^ found greater public support for public health interventions which specifically targeted children and older people than those targeting the entire population. Moreover, people who believed that low‐income populations were to blame for health inequalities were less supportive of welfare interventions, be that targeted (ie those for children or older people) or population level. More recently (2019), an online survey with adults resident in England found levels of acceptability for nudge‐based (eg altering portion sizes) and tax‐based (eg increasing the price of a product) policies were dependent on the policy and the target behaviour; support for a policy was found to decrease as levels of engagement in the behaviour targeted by the policy increased.^27^ For example, those who drank more units of alcohol in a week were less accepting of a policy targeting alcohol. Moreover, one study which conducted nationally representative surveys in six European countries found the UK and Italy (compared to Denmark, France, Germany and Hungary) to be the most disposed towards nudge‐based public health policies.^22^ Overall, however, there is a dearth of nationally representative surveys on the public's views and appetite for public health measures in the academic literature.

In Wales, public engagement in decision‐making is an integral part of policy formation with novel legislation championing the public as a key independent stakeholder in the development of government policies and activities.^28^ Thus, using co‐design and the principles of participatory design,^29^ we conducted a nationally representative survey of the public's views on current public health issues in Wales to engage individuals who would be affected by policy changes in the decision‐making process. The purpose of the study was to co‐create the long‐term strategy for the national public health agency. Here, we report the public's acceptability of 12 public health statements, examining differences across participant characteristics including socio‐demographics, health behaviours, health status and well‐being.

## 2 METHODOLOGY

### 2.1 Ethical approval

Ethical approval was obtained from Bangor University Healthcare and Medical Sciences Academic Ethics Committee. Approval to conduct the study was also received from Public Health Wales Research and Development Office. Interviewers followed the Market Research Society Code of Conduct and adhered to the Declaration of Helsinki. All participants provided informed verbal consent to take part.

### 2.2 Survey sample

A cross‐sectional nationally representative household survey of residents aged 16+ years living in Wales UK was conducted in September and October 2017. The target sample size was 1000. A random probability sampling approach was employed, stratified by Health Board Area, and Lower Super Output Areas (geographical areas with a population mean of 1600) sampled per deprivation quintile to ensure the sample matched the population of Wales. Lower Super Output Areas were categorized into deprivation quintiles based on the Welsh Index of Multiple Deprivation 2014^30^ (a composite deprivation measure inclusive of income, employment, health and qualification measures). One hundred Lower Super Output Areas were sampled proportionate with each Health Board Area's population and across each deprivation quintile. Approximately 30 addresses were randomly selected in each Lower Super Output Area with addresses identified from the post‐code address file.

### 2.3 Data collection

Letters were issued to randomly selected households (N = 3041) to provide residents with information on the study and the opportunity to withdraw by phone or email. A total of 182 (6.0%) households opted out at this stage. Households that had not opted out were visited by trained researchers between 9AM and 8PM, across all days of the week. Each household was visited a maximum of five times. Upon contact with residents, researchers presented a letter of authority and provided a second opportunity to withdraw from the study. In order to identify participants, the inclusion criteria applied were (a) resident in Wales; (b) aged 16+ years; and (c) cognitively able to participate (defined as at the door the participant was able to provide informed consent, that is mental capacity, competence and comprehension to understand the research, appreciate what participation would entail and ability to give adequate informed consent to research).

Only one individual from each household was able to participate and in houses with multiple occupants, the occupant with the next birthday was selected to participate.

Recruitment in each LSOA continued until the target sample was met. This resulted in contact being made with 1673 households. Of those, 358 households declined to participate and four were ineligible, resulting in 1003 completed interviews and an overall completion rate of 60% (1003 agreeing from 1673). Analyses were undertaken with 1001 individuals for whom all socio‐demographic information (ie gender, age, deprivation) was available.

### 2.4 Questionnaire

The questionnaire was designed drawing on previous public opinion surveys^14^, ^31^ to capture the public's views on (a) what they identify as the largest contributors to poor health and well‐being; (b) what public health issues require public service action; (c) where they source health information; and (d) their acceptability of different public health statements. Analyses here focus on the latter outcome. The questionnaire was piloted for understanding and timing, and adjusted according to preliminary feedback.

Participants were asked to self‐report whether they agreed or disagreed with 12 public health statements using a 5‐point Likert scale (see Table 1). The 12 public health statements were selected based on public health priorities in Wales,^33^, ^34^ and questions included related to public health surveys in other countries. A range of demographic questions were included such as participant age, gender, and whether they have children aged under 18 years. Deprivation was derived from the Welsh Index of Multiple Deprivation.^28^ To explore how health and health‐related behaviours influence attitudes towards public health policies and intervention, a series of health‐related questions were asked. In summary, topics covered (a) self‐rated general health status (categorized into low, moderate and high), (b) fruit and vegetable consumption (0‐2, 3‐4, 5+ portions per day), (c) physical activity levels (0‐1, 2‐4, 5+ days per week), (d) binge drinking frequency (regularly, occasionally, never/do not drink) and (e) smoking status (current smoker, ex‐smoker, never smoked). Three questions on well‐being measured whether the individual felt: (a) safe and secure in their local community, (b) optimistic about life and (c) isolated in their community. Using a 5‐point Likert scale, participants were asked whether they agreed, had no opinion, or disagreed with each statement. Full questions and response categories are shown online in Table S1.

**Table 1:** Proportion who agreed/strongly agreed, neither agreed nor disagreed, and disagreed/strongly disagreed with each public health statement (N = 1001)

Public health statement	Agreed/strongly agreed (%)	Neither agreed nor disagreed (%)	Disagreed/strongly disagreed (%)
1. The NHS should spend less on treating illness and more on preventing it	52.9 (49.8‐56.1)	32.1 (29.2‐35.1)	15.0 (12.8‐17.3)
2. Advertising of unhealthy foods to children should be banned to reduce childhood obesity	71.8 (68.9‐74.6)	15.6 (13.4‐18.0)	12.6 (10.6‐14.8)
3. Advertising of alcohol should be banned to reduce alcohol problems	48.7 (45.5‐51.8)	26.1 (23.4‐28.9)	25.3 (22.6‐28.1)
4. Healthy foods should cost a bit less and unhealthy foods a bit more	82.9 (80.4‐85.2)	11.7 (9.8‐13.8)	5.4 (4.1‐7.0)
5. Companies and individuals should be made to adopt behaviours to reduce climate change	66.2 (63.2‐69.2)	24.7 (22.0‐27.5)	9.1 (7.4‐11.0)
6. I support 20mph speed limits where they will reduce road traffic injuries	77.3 (74.6‐79.9)	11.3 (9.4‐13.4)	11.4 (9.5‐13.5)
7. I would like more public information campaigns on how to live a healthier life	47.0 (43.8‐50.1)	25.2 (22.5‐28.0)	27.9 (25.1‐30.8)
8. Schools should teach children more about how to live a healthy life	86.9 (84.7‐88.9)	7.3 (5.8‐9.1)	5.8 (4.4‐7.4)
9. Parents should be given professional advice on how to raise their children well	51.6 (48.5‐54.8)	24.9 (22.2‐27.7)	23.5 (20.9‐26.2)
10. A safe and loving childhood is essential to becoming a healthy adult	87.6 (85.4‐89.6)	8.3 (6.7‐10.2)	4.1 (3.0‐5.5)
11. Employers should do more to look after their workers’ health	75.5 (72.7‐78.2)	16.3 (14.0‐18.7)	8.2 (6.6‐10.1)
12. People should keep themselves healthy, it's not the job of public services	78.9 (76.3‐81.4)	15.3 (13.1‐17.7)	5.8 (4.4‐7.4)

### 2.5 Statistical analysis

Statistical analyses were conducted using SPSS v24. The proportion who agreed/strongly agreed, had no opinion or disagreed/strong disagreed was calculated for each statement. Consistent with the focus of the paper,^21^ where analyses examined agreement, proportions were then dichotomized into those who agreed (agreed/strongly agreed) and those who did not agree (disagreed/strong disagreed/neither agreed nor disagree). Chi‐square was used for initial bivariate analyses to assess the relationship between agreement and participant characteristics (socio‐demographics [eg age, gender], health behaviours, health status and well‐being). Using multivariate logistic regression (backward conditional method), we examined independent associations (*P* < .05) between agreement with each statement and participant characteristics, controlling for socio‐demographics (ie age, gender, deprivation and children) throughout.

## 3 RESULTS

Ten of the 12 public health statements were supported (agreed/strongly agreed) by the majority (>50%) of participants (Table 1). Binary relationships between each statement and participant characteristics are shown online in Tables S2‐S4. Significant independent associations found among participant characteristics in the multivariate analyses of key statements are described below.

The statement receiving the greatest level of support was ‘*a safe and loving childhood is essential to becoming a healthy adult’*; 87.6% agreed with this statement (4.1% disagreed; 8.3% had no opinion; Table 1). In a logistic regression analysis for this statement, agreement was found to be independently associated with feeling safe and secure, feeling optimistic about life and never binge drinking (or drinking at all); individuals who reported feeling safe and secure were three times more likely to agree with the statement than those who did not (or had no opinion; Tables 2, 3, 4). Despite recognition of the importance of safe and loving childhood environments, only half of participants (51.6%) agreed that ‘*parents should be given professional advice on how to raise their children well’,* while a quarter (23.5%) disagreed (Table 1). Agreement for this statement was independently associated with being male, not having children aged under 18 years, having lower general health, high fruit and vegetable consumption (5+ portions) and feeling isolated; those meeting fruit and vegetable consumption guidelines (5+ portions daily) were nearly twice as likely to agree than those who consumed very little (Tables 2, 3, 4). Most participants (86.9%) agreed that ‘s*chools should teach children more about how to live a healthy life’* (5.8% disagreed; Table 1)*,* with males being significantly more likely than females to agree and those who felt safe and secure were nearly twice as likely to agree than those who did not (Tables 2 and 4).

**Table 2:** Logistic regression analysis (backward method) of those who agreed/strongly agreed with each public health statements and their association with socio‐demographics

		Age (y)				Gender	Deprivation quintile					Children
		16‐29	30‐49	50‐69	70+	Female^a^	1 (least)	2	3	4	5 (most)	Yes^a^
1. The NHS should spend less on treating illness and more on preventing it	AOR (95% CI)	*Ref*					*Ref*					
	*P*				ns	ns					ns	ns
2. Advertising of unhealthy foods to children should be banned to reduce childhood obesity	AOR (95% CI)		1.46 (0.96‐2.22)	2.82 (1.83‐4.34)	2.30 (1.47‐3.60)	1.41 (1.07‐1.87)						
	*P*	<.001	.080	<.001	<.001	.016					ns	ns
3. Advertising of alcohol should be banned to reduce alcohol problems	AOR (95% CI)		0.80 (0.53‐1.22)	1.18 (0.79‐1.76)	1.79 (1.17‐2.74)	1.52 (1.18‐1.95)						
	*P*	<.001	.300	.421	.007	.001					ns	ns
4. Healthy foods should cost a bit less and unhealthy foods a bit more	AOR (95% CI)					1.80 (1.29‐2.52)		1.98 (1.13‐3.49)	0.79 (0.49‐1.26)	1.80 (1.03‐3.12)	1.11 (0.67‐1.84)	
	*P*				ns	<.001	.003	.018	.317	.038	.677	ns
5. Companies and individuals should be made to adopt behaviours to reduce climate change	AOR (95% CI)		1.47 (0.93‐2.31)	1.13 (0.73‐1.75)	0.58 (0.37‐0.91)							0.65 (0.45‐0.95)
	*P*	<.001	.100	.581	.018	ns					ns	.025
6. I support 20mph speed limits where they will reduce road traffic injuries	AOR (95% CI)		1.96 (1.24‐3.10)	1.82 (1.17‐2.82)	2.70 (1.65‐4.41)	1.86 (1.38‐2.52)						
	*P*	.001	.004	.008	<.001	<.001					ns	ns
7. I would like more public information campaigns on how to live a healthier life	AOR (95% CI)											
	*P*				ns	ns					ns	ns
8. Schools should teach children more about how to live a healthy life	AOR (95% CI)					0.642 (0.44‐0.94)						
	*P*				ns	.023					ns	ns
9. Parents should be given professional advice on how to raise their children well	AOR (95% CI)					0.76 (0.59‐0.97)						0.66 (0.50‐0.88)
	*P*				ns	.028					ns	.004
10. A safe and loving childhood is essential to becoming a healthy adult	AOR (95% CI)											
	*P*				ns	ns					ns	ns
11. Employers should do more to look after their workers’ health	AOR (95% CI)							1.14 (0.74‐1.76)	1.24 (0.81‐1.91)	1.66 (1.05‐2.61)	2.33 (1.44‐3.77)	
	*P*				ns	ns	.006	.547	.320	.030	.001	ns
12. People should keep themselves healthy, it's not the job of public services	AOR (95% CI)		1.28 (0.81‐2.01)	1.91 (1.22‐3.01)	2.93 (1.75‐4.90)							
	*P*	<.001	.287	.005	<.001	ns					ns	ns

Abbreviations: 95% CI, 95% confidence intervals; AOR, adjusted odds ratio; ns, non‐significant (*P* > .05); Ref, reference category and *P*‐values presented in Ref columns relate to testing of the overall contribution of each variable to the model; Other *P*‐values compare individual categories with the reference category.

^a^ For binary variables, reference categories were male (for gender) and no (for children).

**Table 3:** Logistic regression analysis of those who agreed/strongly agreed with each public health statements and their association with health behaviours (after controlling for socio‐demographics)

		Physical activity (days)			Fruit and vegetable (portions)			Binge drinking frequency			Smoking status		
		0‐1	2‐3	5+	0‐2	3‐4	5+	Regularly	Occasionally	Never	Current	Ex‐smoker	Never
1. The NHS should spend less on treating illness and more on preventing it	AOR (95% CI)	*Ref*			*Ref*	1.01 (0.76‐1.35)	1.44 (1.03‐2.02)	*Ref*			*Ref*		
	*P*			ns	.051	.960	.032			ns			ns
2. Advertising of unhealthy foods to children should be banned to reduce childhood obesity	AOR (95% CI)					1.30 (0.94‐1.79)	1.77 (1.19‐2.63)						
	*P*			ns	.017	.111	.005			ns			ns
3. Advertising of alcohol should be banned to reduce alcohol problems	AOR (95% CI)		0.66 (0.49‐0.89)	0.82 (0.59‐1.15)					0.90 (0.58‐1.41)	1.71 (1.10‐2.64)		1.63 (1.14‐2.34)	1.66 (1.18‐2.33)
	*P*	.025	.007	.246			ns	<.001	.648	.016	.009	.008	.004
4. Healthy foods should cost a bit less and unhealthy foods a bit more	AOR (95% CI)											1.88 (1.19‐2.97)	1.68 (1.10‐2.57)
	*P*			ns			ns				.015	.007	.016
5. Companies and individuals should be made to adopt behaviours to reduce climate change	AOR (95% CI)					1.55 (1.14‐2.11)	1.81 (1.26‐2.61)		1.51 (0.97‐2.37)	1.95 (1.25‐3.05)			
	*P*			ns	.002	.006	.001	.012	.070	.003			ns
6. I support 20mph speed limits where they will reduce road traffic injuries	AOR (95% CI)												
	*P*			ns			ns			ns			ns
7. I would like more public information campaigns on how to live a healthier life	AOR (95% CI)												
	*P*			ns			ns			ns			ns
8. Schools should teach children more about how to live a healthy life	AOR (95% CI)												
	*P*			ns			ns			ns			ns
9. Parents should be given professional advice on how to raise their children well	AOR (95% CI)					1.28 (0.95‐1.72)	1.85 (1.31‐2.61)						
	*P*			ns	.002	.105	<.001			ns			ns
10. A safe and loving childhood is essential to becoming a healthy adult	AOR (95% CI)								1.12 (0.63‐2.00)	1.89 (1.06‐3.39)			
	*P*			ns			ns	.017	.699	.032			ns
11. Employers should do more to look after their workers’ health	AOR (95% CI)					1.29 (0.92‐1.81)	2.05 (1.35‐3.11)						
	*P*			ns	.003	.134	.001			ns			ns
12. People should keep themselves healthy, it's not the job of public services	AOR (95% CI)		1.55 (1.08‐2.21)	1.86 (1.22‐2.83)									
	*P*	.006	.017	.004			ns			ns			ns

Abbreviations: 95% CI, 95% confidence intervals; AOR, adjusted odds ratio; ns, non‐significant (*P* > .05); Ref, reference category and *P*‐values presented in Ref columns relate to testing of the overall contribution of each variable to the model; Other *P*‐values compare individual categories with the reference category.

**Table 4:** Logistic regression analysis of those who agreed/strongly agreed with each public health statements and their association with general health status and well‐being (after controlling for socio‐demographics)

		General health			Well‐being		
		Moderate	Low	High	Felt safe/secure^a^	Felt optimistic^a^	Felt isolated^a^
1. The NHS should spend less on treating illness and more on preventing it	AOR (95% CI)	*Ref*	0.69 (0.51‐0.92)	1.06 (0.78‐1.45)			
	*P*	.014	.012	.714	ns	ns	ns
2. Advertising of unhealthy foods to children should be banned to reduce childhood obesity	AOR (95% CI)					1.75 (1.21‐2.52)	
	*P*			ns	ns	0.003	ns
3. Advertising of alcohol should be banned to reduce alcohol problems	AOR (95% CI)						1.42 (1.01‐1.98)
	*P*			ns	ns	ns	0.043
4. Healthy foods should cost a bit less and unhealthy foods a bit more	AOR (95% CI)				1.79 (1.14‐2.81)	1.78 (1.15‐2.75)	
	*P*				0.011	0.010	ns
5. Companies and individuals should be made to adopt behaviours to reduce climate change	AOR (95% CI)				1.79 (1.24‐2.59)		
	*P*			ns	.002	ns	ns
6. I support 20mph speed limits where they will reduce road traffic injuries	AOR (95% CI)					1.76 (1.20‐2.59)	
	*P*			ns	ns	0.004	ns
7. I would like more public information campaigns on how to live a healthier life	AOR (95% CI)		1.56 (1.17‐2.10)	1.06 (0.77‐1.45)		1.45 (1.02‐2.07)	
	*P*	.008	.003	.725	ns	.040	ns
8. Schools should teach children more about how to live a healthy life	AOR (95% CI)				1.92 (1.22‐3.02)		
	*P*			ns	.005	ns	ns
9. Parents should be given professional advice on how to raise their children well	AOR (95% CI)		0.64 (0.47‐0.86)	1.00 (0.73‐1.38)			1.45 (1.03‐2.04)
	*P*	.006	.003	.991	ns	ns	.035
10. A safe and loving childhood is essential to becoming a healthy adult	AOR (95% CI)				3.01 (1.91‐4.73)	1.63 (1.01‐2.62)	
	*P*			ns	<.001	.045	ns
11. Employers should do more to look after their workers’ health	AOR (95% CI)					1.91 (1.31‐2.78)	
	*P*			ns	ns	.001	ns
12. People should keep themselves healthy, it's not the job of public services	AOR (95% CI)					2.12 (1.45‐ 3.10)	
	*P*			ns	ns	<.001	ns

Abbreviations: 95% CI, 95% confidence intervals; AOR, adjusted odds ratio; ns, non‐significant (*P* > .05); Ref, reference category and *P*‐values presented in Ref columns relate to testing of the overall contribution of each variable to the model; Other *P*‐values compare individual categories with the reference category.

^a^ For binary variables, reference category did not agree (for safe/secure, optimistic, isolated).

Just over half of participants (52.9%) agreed that *the ‘National Health Service (NHS) should spend less on treating illness and more on preventing it’* (15.0% disagreed; Table 1), with agreement independently associated with better general health and high fruit and vegetable consumption (Tables 3, 4). However, less than half of participants (47.0%) agreed that they *‘would like more public information campaigns on how to live a healthier life’,* and over a quarter (27.9%) disagreed (Table 1). Individuals with low general health and those who reported feeling optimistic were both around 1.5 times more likely to agree than those with moderate general health and not feeling optimistic, respectively (Table 4).

Almost eight in 10 participants (78.9%) agreed that ‘*people should keep themselves healthy, it's not the job of public services’* (5.8% disagreed; Table 1). Agreement was independently associated with being aged 50+ years, physically active (2+ days) and feeling optimistic; individuals aged 70+ years were nearly three times more likely to agree with the statement than 16‐ to 29‐year‐olds (Tables 2, 3, 4). Moreover, three quarters (75.5%) agreed that ‘*employers should do more to look after their workers’ health’,* while 8.2% disagreed (Table 1). Agreement was independently associated with greater residential deprivation, high fruit and vegetable consumption and feeling optimistic; those living in the most deprived area were more than twice as likely to agree compared to those living in the least deprived area (Tables 2, 3, 4). Two‐thirds (66.2%) agreed that ‘*companies and individuals should be made to adopt behaviours to reduce climate change’*; only 9.1% disagreed (Table 1)*.* Agreement was independently associated with high fruit and vegetable consumption, never binge drinking and feeling safe and secure, while those aged 70+ years and people with children under 18 years were less likely to agree (Tables 2, 3, 4). People who do not binge drink were nearly twice as likely to agree than those who binge drink regularly (Table 3).

The majority of participants agreed that ‘*healthy foods should cost a bit less and unhealthy foods a bit more’* (82.9%; 5.4% disagreed), that they *‘support 20 mile per hour (mph) speed limits where they will reduce road traffic injuries’* (77.3%; 11.4% disagreed) and that ‘*advertising of unhealthy foods to children should be banned to reduce childhood obesity’* (71.8%; 12.6% disagreed; Table 1). Agreement for all three statements was independently associated with being female and being optimistic, while the youngest age group were least likely to agree with the latter two statements (speed limits and advertising ban; Tables 2 and 4). Agreement with banning junk food advertisements was also independently associated with high fruit and vegetable consumption, while agreement with healthy foods costing less was independently associated with not smoking and feeling safe and secure and optimistic, and varied with deprivation (Tables 2, 3, 4). Moreover, people living in deprivation quintile 2 (an affluent area) were nearly twice as likely to agree that healthy foods should cost less than those living in deprivation quintile 1 (the most affluent area; Table 2). Nearly half of the participants (48.7%) agreed that ‘*advertising of alcohol should be banned to reduce alcohol problems’*, while a quarter (25.3%) disagreed (Table 1). Agreement was independently associated with being female, 70+ years old, never binge drinking, not smoking and being physically inactive (Tables 2, 3). People who never binge drink were 1.7 times more likely to agree with the statement than those who regularly binge drink (Table 3).

## 4 DISCUSSION

The current study aimed to capture a Welsh national perspective on public health policy and interventions across 12 public health areas, and to identify differences across socio‐demographic groups, health‐related behaviours, general health and well‐being. Despite an awareness that co‐creating policy can lead to greater acceptance among the public when implemented, and research undertaken to investigate opinions on specific individual policies such as sugar‐sweetened beverage tax,^35^ a paucity of research has been undertaken to examine public acceptability of a broad range public health topics within a nationally representative sample. This study identified that people in Wales have an appetite for public health intervention in order to improve population health and well‐being, with more than half of the population aged 16+ years in favour of 10 out of 12 public health statements (see Table 1).

A key finding of this study is that those who engage in unhealthy behaviours were least supportive of public health measures (eg 39.8% of binge drinkers agreed alcohol adverts should be banned; see Tables 3, 4), thus identifying a cohort of individuals who are more resistant to interventions. Understanding levels of support for different policy measures and which groups are more or less likely to agree with such policies can inform how receptive residents will be to new policy measures. The public health statements which received the highest levels of public support included those focused on children and nutrition. This reflects similar findings elsewhere in which the authors attributed the finding to the interventions being explicitly health focused (eg nutrition) and that the public identify children as a deserving population group.^21^ In our study, the statement receiving the greatest support was that ‘*a safe and loving childhood is essential to becoming a health adult’* (87.6% agreed; see Table 1). This may reflect a recent prioritization in Wales of work to increase awareness of the harmful impacts of adverse childhood experiences 28, 33 and implement action to support early childhood development, in line with national and global policy commitments to ‘ensure the best start in life, leaving no child behind’.^4^, ^5^ Despite broad recognition of the importance of childhood, only half of the participants (51.6%) agreed that ‘*parents should be given professional advice on how to raise their children well’.* Interestingly, individuals without children under 18 years of age were significantly more in favour (54.5%) of professional parenting advice than those who had children aged under 18 years (43.7%). This finding is in contrast to research in the United States which identified that a majority of parent's wished they had more parenting information.^36^ This difference could be due to differences in culture and the provision of support. Further work is required as to how is best to share information with parents and how to improve the public acceptability of the messages delivered.

Differences in acceptability of the 12 individual statements across participant socio‐demographics, health behaviours, general health and well‐being were also found. Males and females were in agreement on the action of seven policies (see Table 2). However, females were significantly more likely to be in favour of banning adverts on junk food and alcohol, the introduction of 20mph speed limits and healthier foods costing less, while males were found to be more supportive of schools teaching children about how to live a healthier life. Female support for policy relating to alcohol and obesity identified here is consistent with previous studies.^7^, ^27^ Researchers have suggested that females are more supportive than males as they are more health conscious.[27, 37] However, further research is required to identify what is driving the difference found between genders. Similar differences were found between age groups (see Table 2). Older cohorts were more supportive than younger cohorts of banning alcohol and junk food adverts (as previously found^7^), the introduction of 20mph speed limits and thinking that people should look after themselves. Younger cohorts were also the most supportive of policy to reduce climate change—consistent with previous research^38^ and highlighting younger cohorts as champions for such change. This invested support is particularly helpful for achieving the SDG of reducing climate change.^5^ Little association was found between agreement of policy measures and deprivation.^27^ The only significant difference identified was that residents of the most deprived areas were more supportive of employers doing more to look after their workers’ health than residents of the least deprived areas. This could be due to individuals living in more deprived areas being in more physically demanding employment, having less flexibility or a greater reliance on employers for support as they may have less support external to work.

Previous literature has shown that people's acceptability of a policy decreases if they engage in the behaviour targeted by the policy.^7^, ^27^ We explored how acceptability differed by levels of physical activity, binge drinking, smoking, and fruit and vegetable consumption. People who engaged in an unhealthy lifestyle (across all four behaviours) were typically less likely to support public health statements (see Table 3). Of the statements explored which related the behaviours measured, those who consumed the least fruit and vegetables were significantly less likely to agree that junk food adverts should be banned than those who consumed the most; and regular binge drinkers were significantly less likely to agree that alcohol adverts should be banned than none binge drinkers. While such views can result from people's self‐interests,^7^ individuals reporting unhealthier behaviours were also found to be less supportive of other public health statements measures. For instance, those reporting low fruit and vegetable consumption were also less likely to agree with statements that the NHS should spend more on prevention and less on treatment; that companies and individuals should change behaviours to reduce climate change; that parents should be given parenting advice; and that employers should do more to improve employee health. Those who engaged in the lowest level of physical activity were less likely to agree that people should look after themselves and current smokers were less likely than non‐smokers to agree that healthy foods should cost a little less and unhealthy foods a little more. Such findings may suggest that people who engage in health‐harming behaviours are less health conscious in general. Binge drinkers were less likely to agree that a safe and loving childhood is essential to becoming a healthy adult. Although not measured here, harmful alcohol use is associated with adverse childhood experiences (eg neglect and abuse)^3^, ^39^, ^40^, ^41^ and links here may reflect those who binge drink having been less likely to experience and consequently value supportive parenting. Results elsewhere suggest the proportion of the population engaging in the four unhealthy behaviours measured in this study (see Table 3) is declining (although not as quickly among individuals from lower socio‐economic status backgrounds). Our results suggest that such a reduction may be accompanied by increased support for public health policies to tackle the same behaviour. Longitudinal data should be used to examine this hypothesis.

The final participant characteristic we explored was health and well‐being. Optimistic individuals were significantly more in favour for seven of the statements than more pessimistic individuals; all seven statements were prevention‐focused (eg more health campaigns, cost of food, loving childhood; see Table 4). Given that optimistic individuals have been found previously to engage in less unhealthy behaviour and report higher quality of life, it is unsurprising that they would support prevention health measures.^43^, ^44^ Critically, those who reported that they felt safe and secure in their community were significantly more likely to agree with statements on healthy foods costing less, climate change, schools teaching more on health, and the importance of a safe and loving childhood. However, those who reported they felt isolated in their community were significantly more supportive of banning alcohol adverts and parents being given professional advice on child rearing. Recognizing that how people feel in their community can affect their support for broader public health policies can inform multi‐agency working, including health, criminal justice and education sectors.

Overall, our findings suggest that there is public support for public health policies and interventions to promote preventative approaches and help the population to live a healthier life. Those who are optimistic about life are more likely to champion a public health approach. Communicating the effectiveness of policies can increase public support for them,^45^ yet given that our study found levels of support varied by participants’ health behaviours, methods of communication should be developed with this in mind. A one‐size fits all approach is unlikely to work for public health policy, and understanding what drives people's opinions will facilitate the language required and extent to which messaging is needed when targeting specific population groups.^46^ Our results suggest that potentially, if a nation becomes healthier, a greater proportion of people may also be in favour of living healthier and happier lives. Such positive feedback could create a tipping point as a majority in favour of stronger public health policies encourage even more people to move in a healthier direction. Equally, however, in countries where health‐harming behaviours are prevailing or increasing, public health bodies may have more difficulty mustering public support for health improving polices. In their absence, a reduction in public health may occur further increasing resistance to public health policies.

This study is not without limitations. For instance, all data were self‐reported and may be affected by recall bias and/or social desirability bias, where individuals do not disclose accurate reflections of their lifestyle or opinions. Research suggests that opinions on health priorities can alter when people are provided with the opportunity to discuss the issues before making a decision;^47^ this opportunity was not provided and while the initial letter outlined the purpose of the study, it did not divulge the public health topics to be explored. There were relatively few existing surveys to draw questions from, yet where possible questions were derived from validated measures, or adapted from national surveys and the questionnaire was piloted to ensure it suited the target audience. For analyses, participant characteristic questions were collapsed into categories meaning some relationships may have been masked. In addition, while the health behaviour, health status and well‐being measures were selected a‐priori based on hypothesized associations with attitudes on the public health statements, stepwise regression has a number of recognized limitations, especially where variables are highly correlated.^48^ Appendix Table S5 identifies that there is relatively low correlation between the key variables used in our models (all *r* < .279). However, we cannot rule out that some variables excluded from our model on the basis of non‐significance with the variables of interest may be associated with outcome variables in populations outside of our sample.^49^ Importantly, further research is required to validate the relationships identified in these models.

While public health interventions have been implemented for several decades, there is an absence in the literature on their acceptability to the public. Understanding the public's views on public health policies is critical to their development, establishment and uptake, but potentially even more important to their sustainability as governments come and go. Here we identified a positive relationship between supporting public health policies and engaging in health improving behaviours. This raises the possibility of a feedback loop where increases in health behaviours are accompanied by more support for public health policies which themselves facilities further health behaviour gains. More work is required on understanding the interactions between health behaviour and policy support. However, our findings suggest that where population health is allowed to deteriorate, public support for the necessary public health policies to reverse such trends may be even harder to find.

## CONFLICT OF INTEREST

The authors have no competing interests to declare.

## Supporting information

TableS1‐5

## Data Availability

The data that support the findings of this study are available from the corresponding author upon reasonable request.
