# Supplementary material for: Public acceptability of public health policy to improve population health: A population‐based survey
Source: Health Expect. 2020 Apr 24;23(4):802–12. doi: 10.1111/hex.13041 (PMC7495082; doi:10.1111/hex.13041)
Supplement: Supplementary file 1 — TableS1‐5 [file HEX-23-802-s001.docx]

**Table S1.** Single questions asked to obtain socio-demographic and health-related information.

|  | **Measure** | **Question** | **Categories *(responses)*** |
| --- | --- | --- | --- |
| **Socio-demographics** | Age | Please specify your age | 16 – 29 years *(16-17; 18-29)*  30 – 49 years *(30-39; 40-49)*  50 – 69 years *(50-59; 60-69)*  70+ years *(70-79; 80-89; 90+)* |
|  | Gender | What is your gender? | Male  Female  Other |
|  | Children | Do you have children under the age of 18 years? | Yes  No |
| **Health behaviour** | Fruit and vegetable (portions) | On a normal day, how many portions of fruit and vegetables (excluding potatoes) would you usually eat (one portion is roughly one handful)? | 0 – 2 portions *(0; 1; 2)*  3 – 4 portions *(3; 4)*  5+ portions *(5 or more)* |
|  | Physical activity (days) | On how many days each week do you engage in at least 30 minutes physical activity (enough to make you out of breath and sweat)? | 0 – 1 day *(Never; 1 day or less))*  2 – 4 days *(2-4 days)*  5+ days *(5 or more days)* |
|  | Binge  drinking frequency† | In the last year how often have you had 6 or more alcoholic drinks in a single  drinking occasion? | Regularly *(daily; weekly)*  Occasionally *(monthly; less than monthly)*  Never *(never; I don’t drink at all)* |
|  | Smoking  status | In terms of smoking tobacco, which of the following best describes you? | Current *(I smoke daily; I smoke occasionally but not daily)*  Ex-smoker *(I used to smoke but do not smoke at all now)*  Never (*I have never smoked)* |
| **General health** | General  health^‡^ | If 100 is the best state of health you could possibly imagine and 0 is the worst state of health you can imagine, how good or bad is your own health generally? | Low (*0-65; ≤25^th^ percentile*)  Moderate (*66-87; >25^th^- <75^th^ percentile*)  High (*88-100; ≥75^th^ percentile*) |
| **Well-being** | Felt safe and secure | I feel safe and secure in my local community. | Agreed *(agreed; strongly agreed)*  Did not agree *(disagree; strongly disagree; neither agree nor disagree)* |
|  | Felt optimistic | Generally I feel optimistic about life. |  |
|  | Felt Isolated | I often feel isolated in my local community. |  |
|  | †Question drawn from AUDIT C tool; see Bush K, Kivlahan DR, McDonell MB, Fihn SD, Bradley KA. (1998). The AUDIT alcohol consumption questions (AUDIT-C): an effective brief screening test for problem drinking. Ambulatory Care Quality Improvement Project (ACQUIP). Alcohol Use Disorders Identification Test. *Arch Intern Med*, 158 (16): 1789-1795.  ^‡^Question adapted from the EQ-5D-Q; see EuroQol, G. (1990). EuroQol - a new facility for the measurement of health-related quality of life. Health policy (Amsterdam, Netherlands), 16(3), 199. Retrieved from: <https://euroqol.org/publications/key-euroqol-references/>. Response derived from 0-100 Visual analogue scale | | |

**Table S2.** Relationship between those who agreed/strongly agreed with each public health statement and socio-demographics.

|  |  | **Age (years)** | | | | **Gender** | | **Deprivation quintile** | | | | | **Children** | |
| --- | --- | --- | --- | --- | --- | --- | --- | --- | --- | --- | --- | --- | --- | --- |
|  |  | **16 - 29** | **30 - 49** | **50 - 69** | **70+** | **Male** | **Female** | **1 (least)** | **2** | **3** | **4** | **5 (most)** | **No** | **Yes** |
| Sample | % | 13.6 | 27.8 | 33.8 | 24.9 | 42.2 | 57.8 | 20.5 | 19.3 | 20.9 | 19.4 | 20.0 | 73.9 | 26.1 |
| 1. The NHS should spend less on treating illness and more on preventing it | % | 50.7 | 50.4 | 51.2 | 59.4 | 54.9 | 51.4 | 47.3 | 59.6 | 50.7 | 55.7 | 52.0 | 54.2 | 49.4 |
|  | X^2^ | 5.647 | | | | 1.220 | | 7.089 | | | | | 1.758 | |
|  | P | 0.130 | | | | 0.269 | | 0.131 | | | | | 0.185 | |
| 2. Advertising of unhealthy foods to children should be banned to reduce childhood obesity | % | 57.4 | 66.2 | 79.3 | 75.9 | 67.7 | 75.2 | 74.6 | 77.7 | 72.7 | 68.6 | 65.5 | 74.2 | 65.1 |
|  | X^2^ | 29.798 | | | | 6.944 | | 9.176 | | | | | 7.818 | |
|  | P | <0.001 | | | | 0.008 | | 0.057 | | | | | 0.005 | |
| 3. Advertising of alcohol should be banned to reduce alcohol problems | % | 44.9 | 39.6 | 49.4 | 59.8 | 42.7 | 53.6 | 47.3 | 49.7 | 46.4 | 46.9 | 53.0 | 49.9 | 45.2 |
|  | X^2^ | 22.520 | | | | 11.688 | | 2.408 | | | | | 1.673 | |
|  | P | <0.001 | | | | 0.001 | | 0.661 | | | | | 0.196 | |
| 4. Healthy foods should cost a bit less and unhealthy foods a bit more | % | 81.6 | 83.8 | 83.1 | 82.3 | 78.8 | 86.3 | 80.5 | 88.6 | 76.6 | 87.6 | 82.0 | 83.0 | 82.8 |
|  | X^2^ | 0.392 | | | | 10.050 | | 14.388 | | | | | 0.006 | |
|  | P | 0.941 | | | | 0.002 | | 0.006 | | | | | 0.937 | |
| 5. Companies and individuals should be made to adopt behaviours to reduce climate change | % | 65.4 | 70.9 | 70.4 | 55.8 | 66.8 | 65.8 | 65.4 | 67.4 | 65.1 | 66.5 | 67.0 | 66.6 | 65.1 |
|  | X^2^ | 17.410 | | | | 0.124 | | 0.363 | | | | | 0.191 | |
|  | P | 0.001 | | | | 0.725 | | 0.985 | | | | | 0.662 | |
| 6. I support 20mph speed limits where they will reduce road traffic injuries | % | 64.7 | 78.1 | 77.2 | 83.5 | 71.2 | 82.3 | 79.5 | 78.8 | 72.2 | 83.0 | 73.5 | 77.0 | 78.2 |
|  | X^2^ | 17.913 | | | | 17.396 | | 9.075 | | | | | 0.141 | |
|  | P | <0.001 | | | | <0.001 | | 0.059 | | | | | 0.707 | |
| 7. I would like more public information campaigns on how to live a healthier life | % | 49.3 | 43.5 | 51.5 | 43.4 | 46.2 | 47.5 | 48.8 | 43.0 | 40.2 | 52.6 | 50.5 | 47.8 | 44.4 |
|  | X^2^ | 5.664 | | | | 0.169 | | 8.793 | | | | | 0.892 | |
|  | P | 0.129 | | | | 0.681 | | 0.066 | | | | | 0.345 | |
| 8. Schools should teach children more about how to live a healthy life | % | 91.9 | 84.5 | 87.6 | 85.9 | 89.6 | 84.7 | 86.3 | 90.7 | 84.2 | 86.1 | 87.5 | 87.6 | 85.1 |
|  | X^2^ | 4.708 | | | | 5.232 | | 3.979 | | | | | 1.069 | |
|  | P | 0.194 | | | | 0.022 | | 0.409 | | | | | 0.301 | |
| 9. Parents should be given professional advice on how to raise their children well | % | 48.5 | 48.9 | 55.9 | 50.6 | 55.8 | 48.3 | 51.2 | 57.5 | 45.9 | 50.5 | 53.5 | 54.5 | 43.7 |
|  | X^2^ | 3.933 | | | | 5.558 | | 5.781 | | | | | 8.981 | |
|  | P | 0.269 | | | | 0.018 | | 0.216 | | | | | 0.003 | |
| 10. A safe and loving childhood is essential to becoming a healthy adult | % | 86.0 | 86.7 | 87.9 | 89.2 | 86.3 | 88.7 | 90.2 | 88.6 | 86.1 | 90.2 | 83.0 | 87.4 | 88.1 |
|  | X^2^ | 1.099 | | | | 1.342 | | 7.031 | | | | | 0.085 | |
|  | P | 0.777 | | | | 0.247 | | 0.134 | | | | | 0.771 | |
| 11. Employers should do more to look after their workers’ health | % | 83.1 | 76.3 | 75.4 | 70.7 | 73.7 | 77.0 | 69.3 | 72.0 | 73.7 | 78.9 | 84.0 | 74.9 | 77.4 |
|  | X^2^ | 7.449 | | | | 1.529 | | 14.949 | | | | | 0.668 | |
|  | P | 0.059 | | | | 0.216 | | 0.005 | | | | | 0.414 | |
| 12. People should keep themselves healthy, it’s not the job of public services | % | 69.1 | 74.1 | 81.1 | 86.7 | 77.4 | 80.1 | 75.1 | 85.0 | 78.0 | 78.4 | 78.5 | 79.3 | 77.8 |
|  | X^2^ | 21.841 | | | | 1.096 | | 6.197 | | | | | 0.277 | |
|  | P | <0.001 | | | | 0.295 | | 0.185 | | | | | 0.598 | |

**Table S3.** Relationship between those who agreed/strong agreed with each public health statement and health behaviours.

|  |  | **Physical activity (days)** | | | **Fruit and vegetable (portions)** | | | **Binge drinking frequency** | | | **Smoking status** | | |
| --- | --- | --- | --- | --- | --- | --- | --- | --- | --- | --- | --- | --- | --- |
|  |  | **0 - 1** | **2 - 4** | **5+** | **0 - 2** | **3 - 4** | **5+** | **Regularly** | **Occasionally** | **Never** | **Current** | **Ex-smoker** | **Never** |
| Sample | % | 40.6 | 36.2 | 23.3 | 32.0 | 43.3 | 24.8 | 10.8 | 37.0 | 52.2 | 22.0 | 31.6 | 46.5 |
| 1. The NHS should spend less on treating illness and more on preventing it | % | 55.4 | 55.4 | 48.1 | 50.6 | 50.8 | 59.7 | 50.0 | 53.5 | 53.2 | 48.6 | 56.6 | 52.5 |
|  | X^2^ | 2.901 | | | 5.997 | | | 0.433 | | | 3.481 | | |
|  | P | 0.234 | | | 0.050 | | | 0.805 | | | 0.181 | | |
| 2. Advertising of unhealthy foods to children should be banned to reduce childhood obesity | % | 71.7 | 73.8 | 69.1 | 64.7 | 72.7 | 79.4 | 73.1 | 67.8 | 74.4 | 62.7 | 73.7 | 74.8 |
|  | X^2^ | 1.528 | | | 15.337 | | | 4.686 | | | 11.655 | | |
|  | P | 0.466 | | | <0.001 | | | 0.096 | | | 0.003 | | |
| 3. Advertising of alcohol should be banned to reduce alcohol problems | % | 54.2 | 43.1 | 50.8 | 48.1 | 47.8 | 50.8 | 39.8 | 38.6 | 57.6 | 37.7 | 51.6 | 51.8 |
|  | X^2^ | 9.551 | | | 0.620 | | | 34.782 | | | 13.474 | | |
|  | P | 0.008 | | | 0.733 | | | <0.001 | | | 0.001 | | |
| 4. Healthy foods should cost a bit less and unhealthy foods a bit more | % | 81.0 | 85.6 | 82.0 | 81.6 | 82.2 | 85.9 | 73.1 | 84.9 | 83.6 | 76.4 | 85.4 | 84.3 |
|  | X^2^ | 3.050 | | | 2.109 | | | 8.418 | | | 8.723 | | |
|  | P | 0.218 | | | 0.348 | | | 0.015 | | | 0.013 | | |
| 5. Companies and individuals should be made to adopt behaviours to reduce climate change | % | 62.6 | 70.4 | 66.1 | 59.1 | 68.6 | 71.4 | 57.4 | 66.8 | 67.7 | 59.5 | 66.5 | 69.2 |
|  | X^2^ | 5.316 | | | 11.361 | | | 4.301 | | | 6.296 | | |
|  | P | 0.070 | | | 0.003 | | | 0.116 | | | 0.043 | | |
| 6. I support 20mph speed limits where they will reduce road traffic injuries | % | 80.0 | 76.2 | 74.2 | 70.9 | 81.3 | 78.6 | 76.9 | 74.9 | 79.2 | 70.9 | 82.6 | 76.8 |
|  | X^2^ | 3.217 | | | 11.575 | | | 2.294 | | | 10.250 | | |
|  | P | 0.200 | | | 0.003 | | | 0.318 | | | 0.006 | | |
| 7. I would like more public information campaigns on how to live a healthier life | % | 50.2 | 45.0 | 44.2 | 46.3 | 45.7 | 50.0 | 41.7 | 45.1 | 49.3 | 46.8 | 45.3 | 48.2 |
|  | X^2^ | 3.013 | | | 1.249 | | | 2.890 | | | 0.646 | | |
|  | P | 0.222 | | | 0.536 | | | 0.236 | | | 0.724 | | |
| 8. Schools should teach children more about how to live a healthy life | % | 85.2 | 90.1 | 85.0 | 87.8 | 85.7 | 87.9 | 89.8 | 87.0 | 86.2 | 87.3 | 87.3 | 86.5 |
|  | X^2^ | 4.930 | | | 1.019 | | | 1.016 | | | 0.163 | | |
|  | P | 0.085 | | | 0.601 | | | 0.602 | | | 0.922 | | |
| 9. Parents should be given professional advice on how to raise their children well | % | 49.8 | 53.9 | 51.5 | 46.3 | 50.8 | 60.1 | 53.7 | 49.5 | 52.8 | 45.5 | 54.4 | 52.7 |
|  | X^2^ | 1.299 | | | 10.918 | | | 1.157 | | | 4.560 | | |
|  | P | 0.522 | | | 0.004 | | | 0.561 | | | 0.102 | | |
| 10. A safe and loving childhood is essential to becoming a healthy adult | % | 87.2 | 89.2 | 85.8 | 84.7 | 88.7 | 89.5 | 83.3 | 84.9 | 90.4 | 83.2 | 88.0 | 89.5 |
|  | X^2^ | 1.612 | | | 3.808 | | | 8.248 | | | 5.484 | | |
|  | P | 0.447 | | | 0.149 | | | 0.016 | | | 0.064 | | |
| 11. Employers should do more to look after their workers’ health | % | 71.7 | 78.5 | 77.7 | 72.2 | 74.4 | 81.9 | 71.3 | 76.2 | 75.9 | 74.5 | 75.9 | 75.7 |
|  | X^2^ | 5.521 | | | 7.619 | | | 1.182 | | | 0.153 | | |
|  | P | 0.063 | | | 0.022 | | | 0.554 | | | 0.927 | | |
| 12 .People should keep themselves healthy, it’s not the job of public services | % | 75.9 | 79.6 | 83.3 | 73.8 | 82.2 | 79.8 | 78.7 | 74.3 | 82.2 | 72.7 | 79.2 | 81.3 |
|  | X^2^ | 5.011 | | | 8.097 | | | 8.120 | | | 6.772 | | |
|  | P | 0.082 | | | 0.017 | | | 0.017 | | | 0.034 | | |

**Table S4.** Relationship between those who agreed/strongly agreed with each public health statement and health status and well-being.

|  |  | **General health** | | | **Well-being** | | |
| --- | --- | --- | --- | --- | --- | --- | --- |
|  |  | **Moderate** | **Low** | **High** | **Felt safe/secure** | **Felt optimistic** | **Felt isolated** |
| Sample | % | 43.4 | 31.6 | 25.1 | 85.4 | 84.6 | 17.6 |
| 1. The NHS should spend less on treating illness and more on preventing it | % | 55.5 | 46.2 | 57.0 | 54.4 | 54.3 | 52.3 |
|  | X^2^ | 8.564 | | | 4.872 | 4.101 | 0.036 |
|  | P | 0.014 | | | 0.027 | 0.043 | 0.851 |
| 2. Advertising of unhealthy foods to children should be banned to reduce childhood obesity | % | 73.0 | 68.7 | 73.7 | 73.8 | 73.8 | 72.4 |
|  | X^2^ | 2.309 | | | 11.277 | 10.470 | 0.036 |
|  | P | 0.315 | | | 0.001 | 0.001 | 0.850 |
| 3. Advertising of alcohol should be banned to reduce alcohol problems | % | 45.4 | 51.6 | 50.6 | 49.8 | 49.8 | 56.9 |
|  | X^2^ | 3.313 | | | 3.230 | 3.025 | 5.731 |
|  | P | 0.191 | | | 0.072 | 0.082 | 0.017 |
| 4. Healthy foods should cost a bit less and unhealthy foods a bit more | % | 84.8 | 82.0 | 80.9 | 84.7 | 84.8 | 82.2 |
|  | X^2^ | 2.019 | | | 12.838 | 13.341 | 0.080 |
|  | P | 0.364 | | | <0.001 | <0.001 | 0.777 |
| 5. Companies and individuals should be made to adopt behaviours to reduce climate change | % | 65.4 | 63.3 | 71.3 | 67.8 | 67.5 | 67.8 |
|  | X^2^ | 4.244 | | | 6.731 | 4.152 | 0.236 |
|  | P | 0.120 | | | 0.009 | 0.042 | 0.627 |
| 6. I support 20mph speed limits where they will reduce road traffic injuries | % | 78.6 | 77.8 | 74.5 | 78.9 | 79.0 | 79.3 |
|  | X^2^ | 1.575 | | | 8.825 | 8.673 | 0.475 |
|  | P | 0.455 | | | 0.003 | 0.003 | 0.491 |
| 7. I would like more public information campaigns on how to live a healthier life | % | 43.3 | 53.5 | 45.0 | 46.8 | 48.1 | 51.7 |
|  | X^2^ | 8.086 | | | 0.068 | 2.669 | 1.925 |
|  | P | 0.018 | | | 0.795 | 0.102 | 0.165 |
| 8. Schools should teach children more about how to live a healthy life | % | 88.9 | 84.8 | 86.1 | 88.2 | 88.1 | 87.4 |
|  | X^2^ | 2.959 | | | 8.366 | 6.541 | 0.036 |
|  | P | 0.228 | | | 0.004 | 0.011 | 0.849 |
| 9. Parents should be given professional advice on how to raise their children well | % | 55.1 | 45.6 | 53.4 | 52.0 | 52.7 | 56.9 |
|  | X^2^ | 7.013 | | | 0.373 | 2.240 | 2.323 |
|  | P | 0.030 | | | 0.542 | 0.134 | 0.127 |
| 10. A safe and loving childhood is essential to becoming a healthy adult | % | 88.0 | 87.3 | 87.3 | 90.2 | 89.3 | 84.5 |
|  | X^2^ | 0.117 | | | 35.482 | 13.707 | 1.901 |
|  | P | 0.943 | | | <0.001 | <0.001 | 0.168 |
| 11. Employers should do more to look after their workers’ health | % | 74.7 | 76.3 | 76.1 | 76.5 | 77.3 | 78.2 |
|  | X^2^ | 0.316 | | | 2.964 | 9.782 | 0.792 |
|  | P | 0.854 | | | 0.085 | 0.002 | 0.374 |
| 12. People should keep themselves healthy, it’s not the job of public services | % | 80.4 | 75.3 | 80.9 | 80.6 | 81.2 | 75.3 |
|  | X^2^ | 3.627 | | | 9.754 | 17.610 | 1.672 |
|  | P | 0.163 | | | 0.002 | <0.001 | 0.196 |

**Table S5. Spearman rank correlation relationship between the confounding variables**

|  | **Health behaviours** | | | **Well-being measures** | | |
| --- | --- | --- | --- | --- | --- | --- |
|  | **2** | **3** | **4** | **6** | **7** | **8** |
| **Health behaviours** |  |  |  |  |  |  |
| 1. Physical activity | 0.159** | -0.064* | -0.005 |  |  |  |
| 2. Fruit and vegetable |  | 0.072* | 0.256** |  |  |  |
| 3. Binge drinking frequency |  |  | 0.127** |  |  |  |
| 4. Smoking status |  |  |  |  |  |  |
| **Well-being** |  |  |  |  |  |  |
| 5. General health |  |  |  | 0.006 | 0.005 | -0.007 |
| 6. Felt safe/secure |  |  |  |  | 0.279** | -0.176** |
| 7. Felt optimistic |  |  |  |  |  | -0.155** |
| 8. Felt Isolated |  |  |  |  |  |  |
| * p<0.05, ** p <0.001 | | | | | | |
